# Sometimes Small Is Beautiful: Discovery of the Janus Kinases (JAK) and Signal Transducer and Activator of Transcription (STAT) Pathways and the Initial Development of JAK Inhibitors for IBD Treatment

**DOI:** 10.1007/s10620-024-08791-1

**Published:** 2025-01-18

**Authors:** Jonathan D. Kaunitz

**Affiliations:** Medical Service, Greater Los Angeles VAMC and Department of Medicine, David Geffen School of Medicine at UCLA, Los Angeles, CA 90073 USA

**Keywords:** JAK/STAT pathway, JAKininbs, Tofacitinib, Cytokines, Interferons, Interleukins

## Abstract

The Janus kinase/signal transducer and activator of transfection (JAK/STAT) system is comprised of multiple cell surface receptors, receptor tyrosine kinases, and signal transducers that are key components of numerous systems involved in malignancy, inflammation, immune surveillance and development, cellular proliferation, metabolism, differentiation, apoptosis, and hematologic disorders, all of which when disrupted can produce severe disease. Nevertheless, small molecule inhibitors of the four known JAKs, termed JAKinibs, have found therapeutic indications for a broad category of diseases. In this perspective, I will summarize the development of JAK inhibitors, whose origins were in antiquity, with particular attention to their use in treating patients with inflammatory bowel disease (IBD). This perspective is accompanied by a companion publication addressing how JAKinibs have forever altered the landscape of IBD therapy.

## Introduction

Inflammatory bowel disease (IBD), encompassing ulcerative colitis and Crohn disease, are chronic autoimmune diseases that cause considerable morbidity [1]. Although treatments have evolved rapidly in the past two decades, response rates still hover at < 50% even for the best performing treatments [2] with remission rates limited to 20–30% (according to some authors) [3], with durable responses rare.

The IBD therapeutic revolution started with a monoclonal antibody directed against tumor necrosis factor (TNF)-α termed antibody cA2 (now known as infliximab) in 1995 [4], one of the few drugs other than corticosteroids that achieved good efficacy in moderate-to-severe disease. Since then, monoclonal antibodies directed against other immunomodulatory targets have been developed and used clinically. Although monoclonal antibodies have achieved good success and have expanded to other molecular targets, efficacy has remained suboptimal. The next major advance was the recognition that small molecule inhibitors of the Janus kinase (JAK) family had activity against autoimmune conditions such as the autoimmune arthridities [5] and IBD [6]. These drugs thus far have shown an excellent efficacy and safety profile, holding promise for future refinement given the large number of agents currently in development. Unlike the monoclonal antibodies, JAK inhibitors are given orally, holding promise for convenience and eventually for low cost.

In this installment of the “Paradigm Shifts in Perspective” series, I will provide a brief introduction to the basic science advances underlying the development of the JAK inhibitors followed by a review of the history of the clinical development of the first small molecule JAK inhibitors (JAKinibs). This article accompanies a review of the current state of the art for the use of JAK inhibitors in patients with IBD, and the future of this therapeutic area. [7]

One of the primary purposes of this series is to remind the reader that major advances are preceded by decades or even centuries of painstaking research, often in obscure fields that seem to bear little relation to the disease that is eventually treated. It is only through the continued application of innovation, thoughtfulness, and the courage of dedicated scientists to think differently and creatively that these major shifts will continue to occur.

## Historical Context

The discovery of the JAK family can be traced back to the discovery of the interferons (IFNs), arguably the first cytokine identified. The origin of this major discovery is attributed to ancient Greece and Anatolia, where according to the mid-twentieth century infectious disease specialist A. W. Downie [8], in antiquity, smallpox exudates were introduced through small cutaneous scratches in order to confer immunity to subsequent infection in a process termed variolization. This practice spread to England in the early eighteenth century, where it was refined, although still considered risky due to inadvertent transmission of smallpox to the vaccinee. The paradigm shift occurred when Edward Jenner hypothesized that inoculation with exudates from the relatively innocuous disease cowpox (vaccinia) could protect from subsequent infection by smallpox (variola). Groundbreaking studies published by Jenner in 1798 [9] serve as the formal beginning of immunology and the birth of vaccinations, likely the most cost-effective medical intervention to date. This protection from a subsequent viral infection by a previous viral infection was termed “viral interference.”

Experimental data underlying the mechanism of viral interference mostly began accumulating in the 1920s, exhaustively reviewed and summarized by Werner Henle [10], grandson of the eminent German anatomist Jakob Henle, in which he listed and discussed six possible mechanisms underlying the phenomenon of viral interference, mostly based on the observation that inactivated influenza viruses inhibited the growth of live influenza viruses grown in chick embryos. Of these, “prevention of spread of the excluded virus by the inflammatory tissue response induced by the interfering agent” and “antiviral activities…of some product resulting from the primary infection” were later confirmed to be correct. Although many studies of viral interference had been published, the use of the chick embryo system led the first breakthrough in 1957 by Isaacs and Lindemann [11], who are credited with the discovery and initial characterization of IFN using pieces of the chorioallantoic membrane from chick embryos that elaborated a fluid that over time inhibited the growth of live influenza virus. Crow et al. [12], however, argue that Nagano and Kojima deserve credit for their 1954 publication [13] identifying interferon based on their studies of UV-irradiated vaccinia virus, identifying a factor in a supernatant that conferred immunity to subsequent vaccinia infection. Subsequent studies more fully characterized the IFNs as a family of molecules responsible for viral interference [14] later identified as key pro-inflammatory cytokines that activated specific membrane receptors, inducing a pro-inflammatory program in the recipient cells, contributory to the pathogenesis of autoimmune diseases such as lupus erythematosus. [12]

The next major advance was the discovery of the protein tyrosine kinases (PTK), enzymes that phosphorylate tyrosine residues on proteins. The discovery of the tyrosine kinases can be traced to 1978, when Collett and Erickson [15] reported that the gene product of the avian sarcoma virus (ASV) termed p60^src^ phosphorylated a protein in ASV-transformed chick cell lysates. Although these scientists only reported a phosphothreonine product, Tony Hunter and co-workers [16] unambiguously identified a phosphotyrosine product of p60^src^ in 1980 that was not only the first identified tyrosine kinase, but also the first reported oncogene, now known collectively as the *Src* family kinases, a subset of the more than 50 tyrosine kinases thus far identified, which roughly belong to two major classes: the receptor tyrosine kinases (RTK) in which a transmembrane growth factor receptor possesses the TK activity, and the non-receptor TK family, which can be associated with transmembrane receptors. The RTK, due to their overexpression in some tumors and their strong pro-proliferative effects, have seen success as targets of cancer therapy, as have some non-RTK. [17]

## Discovery of the JAK Family

In 1989, Andrew Wilks [18] reported that he had identified two new members of the non-receptor PTK family by a then novel method, the polymerase chain reaction [19]. Further study from Wilks’ laboratory in collaboration with the laboratory of Andrew Ziemiecki [20] in Switzerland revealed that these novel PTK that he named the Janus kinases, after the two-faced Roman god due to the presence of second phosphotransferase located opposite to the first [21]. The two other members of the JAK family, tyrosine kinase (TYK)2 [22] and JAK3 [23] were subsequently cloned by several other laboratories in short order.

**Fig. 1:**
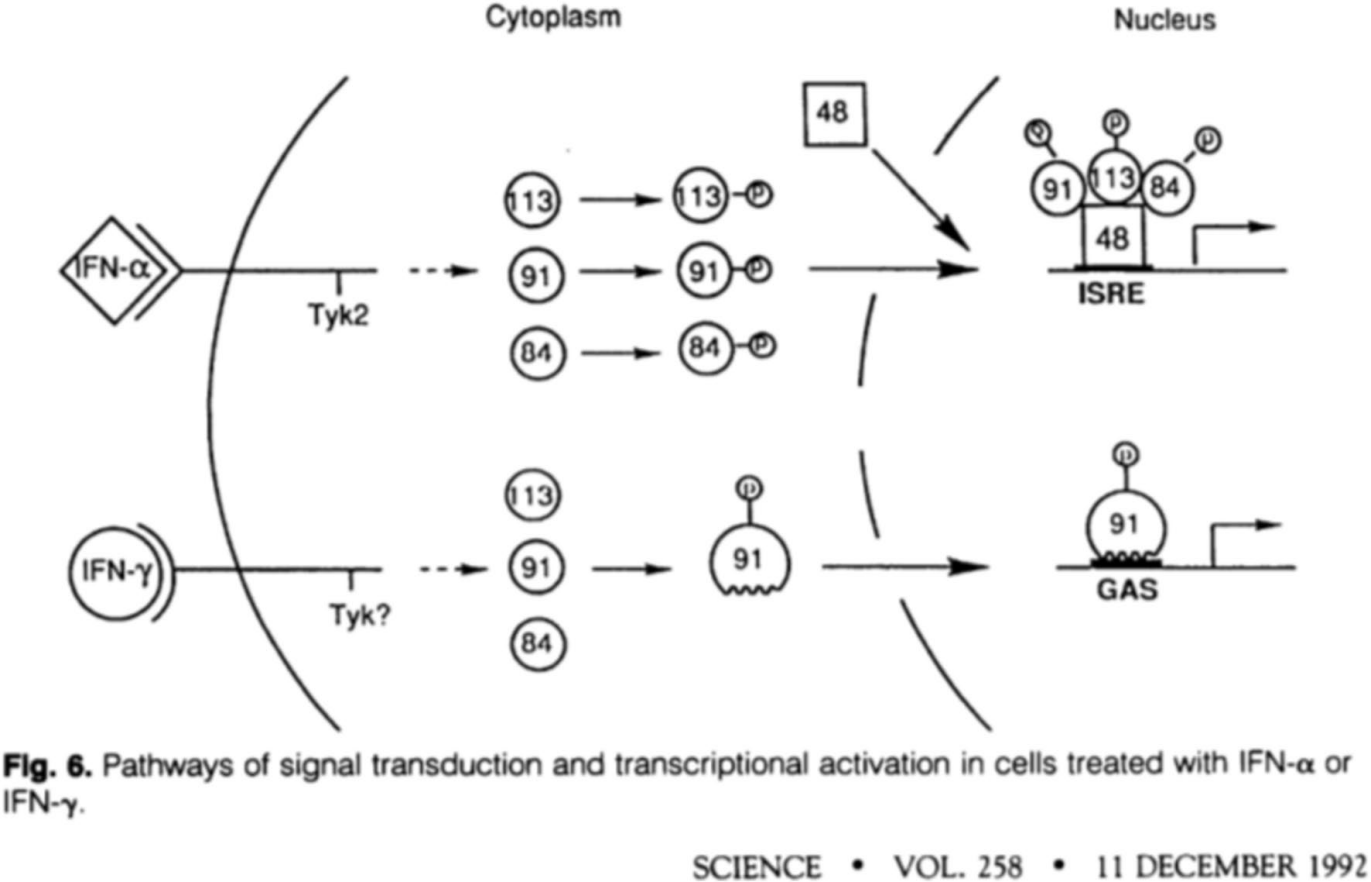
One of the first schematic diagrams of the JAK-STAT signaling system. The numbers 84, 91, and 113 signify the molecular weights of IFN-induced phosphoproteins, which were later ascribed as the following: 91 – STAT1; 113 – STAT2; 84 – STAT3 [29,71]. *ISRE* interferon-stimulated response element, *GAS* γ-activated site. Reprinted with permission from the publisher from Ref. [28]

At about the same time, Knight and Korant [24], working at the research laboratories of the du Pont corporation, reported that exposure of human fibroblast cells to IFN induced the synthesis of four heretofore unidentified protein kinases. Further studies conducted in the 1980s by Jim Darnell’s lab at the Rockefeller University [25] and George Stark’s lab at Stanford and the Imperial Cancer Research Fund in London [26] further identified the kinetics of IFN-induced gene expression and a DNA response element that bound to the induced proteins. Further data supporting the function of these kinases were reported in 1991 from Sandra Pellegrini’s lab at the Institut Pasteur in Paris in collaboration with George Stark in London [27] in which mutant 11,1 cells unresponsive to IFNα recovered responsiveness after transfection with a transcript identified as TYK2, strengthening the link between activation of the cell surface IFN receptor with activation of cellular IFN-responsive genes. In 1992, Ke Shuai [28] in Darnell’s lab reported that one of the IFN-induced proteins, a 91-kD DNA binding protein was tyrosine phosphorylated and then translocated to the nucleus where it bound to a specific DNA binding element. This publication contains an early, if not the first schematic of the JAK-STAT pathway (Fig. 1) in which activation of a transmembrane cytokine receptor by a pro-inflammatory cytokine (in this case IFNs) activates a TK that phosphorylates an IFN-induced protein that then translocates to the nucleus where it binds to a specific DNA binding element. The 91-kD protein was subsequently named signal transducer and activator of transfection (STAT)-1 [29], which Darnell explains arose from a conversation with his late wife Jane who stated that STAT, derived from the Latin *statum* in medical jargon, means immediately, reflecting the rapidity of IFN-induced protein synthesis.

The final (for now) piece of the puzzle was the discovery of the endogenous inhibitory protein families, including the suppressors of cytokine signaling (SOCS), protein inhibitors of activated STATs (PIAS), and the cytoplasmic phosphatase SHP-1, whose inhibition of JAK-STAT-mediated signal transduction was initially recognized in the late 1990s [30].

**Fig. 2:**
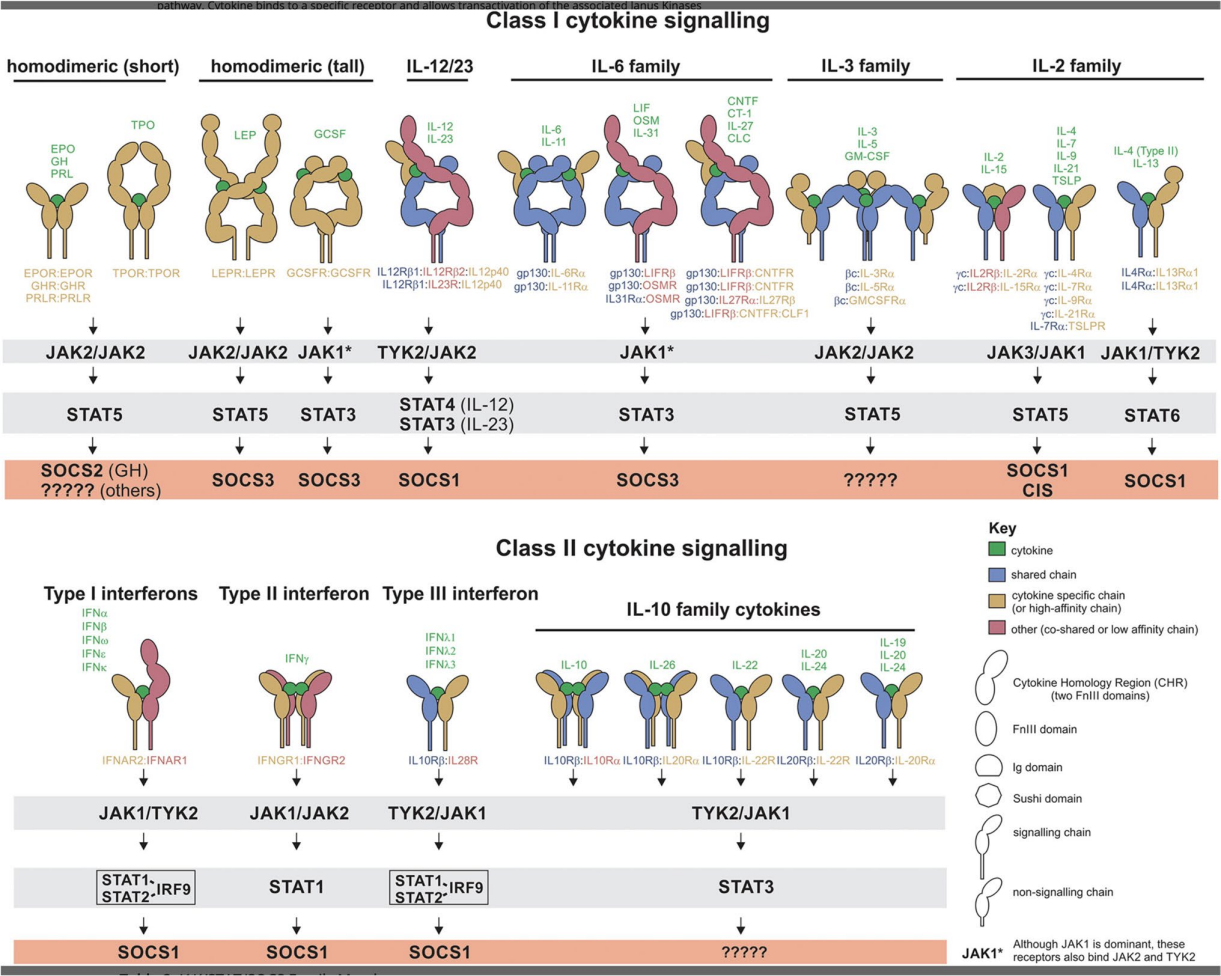
Schematic depiction of the JAK-STAT-SOCS cytokine signal transduction system divided into Class I and Class II cytokine families. Note that the JAK2/TYK2 combination is solely associated with the heterodimeric IL-12/23 receptor. Reprinted with permission from Ref. [68]

The reader is referred to two excellent reviews co-authored by George Stark and by my former undergraduate molecular biology professor Jim Darnell [29,31], chronicling the discovery and evolution of knowledge about the JAK-STAT pathway dating from the mid-1950s, providing a first-hand, blow-by-blow description of the exciting sequence of discoveries that led to what is now termed the JAK-STAT pathway, a major discovery with myriad implications for disease treatment. If you are looking for a detailed account of these discoveries or like to read about the exhilaration accompanying scientific breakthroughs, these articles are highly recommended. A schematic of the currently known members of the JAKS/STAT/SOCS pathway is depicted in Fig. 2.

## Initial development of the JAK inhibitors

### Background

Increasing knowledge of the JAKs indicated that this enzyme family has wide-ranging functions. Transducing the signals from multiple cytokines interacting with numerous receptors, each associated with a combination of JAKs and STATs, suggests that this system has many redundancies and overlaps. Furthermore, genetic deletion or mutations of members of the JAK family, exhaustively listed by Hammarén et al., [32] list a bewildering array of associated diseases, which generally include malignancy, inflammation, immune surveillance and development, proliferation, metabolism, differentiation, apoptosis, and hematologic disorders, leading to the sobering conclusion that JAKs are needed for normal development of the immune, hemopoietic, and cancer surveillance systems among others, that are completely inhibited at one’s peril. Therefore, it was realized relatively early that partial inhibition of a given JAK to selectively tamp down an overactive pathway while avoiding serious adverse effects is likely a successful strategy.

### The First Small Molecule JAK Inhibitors

Considering how central this pathway is to so many key bodily functions, interest rapidly increased in finding drugs that could inhibit this pathway in the hope of developing novel therapies [33]. Among the earliest described, small molecule JAK inhibitors were reported from the Wayne Hughes Institute in Minnesota from the laboratory of Faith Uckun [34]. Using an in vitro kinase assay using recombinant JAK1-3 expressed in Sf21 cells, they reported that “Compound 1,” a dimethoxyquinazoline based on the pharmacophore of other TK inhibitors, selectively inhibited JAK3. The compound, subsequently named WHI-P131 or JANEX-1 [35], displayed antileukemic activity in vitro and was used to prevent experimental graft-versus-host disease [35]. Despite its initial success, the potency and selectivity, in particularly toward other members of the human kinome, of WHI-P131 were later questioned [36], with these compounds never achieving clinical application.

### Application to Myeloproliferative Disorders

The initial success of WHI-P131 likely spurred the search for other selective small molecule JAK inhibitors. One of the most direct connections between the JAK family and human disease was reported in 2005 by several groups [37–39] who discovered that patients with myeloproliferative disorders such as polycythemia vera and myelofibrosis carried a phenylalanine substitution for valine 617 in the JH2 (pseudokinase) domain of JAK 2 that constitutively activated tyrosine phosphorylation activity. This mutation was later confirmed to cause a polycythemia-like disease in murine models [40,41], serving as the target for the first approved JAK inhibitor INC018424, later called ruxolitinib, that was FDA approved in 2011 for myelofibrosis and related diseases and has achieved qualified success over 10 years of clinical use [42,43]. Since this drug inhibits constitutive JAK2 activation, this is the closest approximation that currently exists to a straight line between human disease and JAK inhibition.

### Application to Immune Regulation

Another direction for JAK inhibitor development was as an immunosuppressant, particularly in organ transplant recipients. The contribution of JAKs to immunosuppression is underscored by findings such as those by Russell et al. [44] and Nosaka et al. [45] in 1995 that JAK3 mutation causes severe combined immunodeficiency, a finding that prompted a search for JAK inhibitor-based immunomodulatory drugs. Accordingly, several pharmaceutical companies embarked on development programs for JAK3 inhibitors in the late 1990s. [21,46] Perhaps the most notable was the development of the compound CP-690,550 [36,46] that was discovered through traditional high-throughput screening of a chemical library using conventional medicinal chemistry methods as retrospectively reported by Paul Changelian and a consortium of pharmaceutical and university-based investigators [21,46]. The authors identified related chemical molecules of a core pyrrolopyrimidine structure that was intended to selectively inhibit JAK1 and JAK3, that were implicated in immunomodulation due to their co-expression on the IL-2 receptor and the limitation of JAK3 expression to immune cells. Initially, ~ 800,000 compounds in the Pfizer library were screened using an in vitro assay for JAK3 kinase activity. The identified lead compounds were then screened for JAK inhibition with somewhat arguable selectivity for JAK1 and JAK 3 and importantly minimal activity against the remainder of the human kinome [21] with the initial aim to discover an immunosuppressive drug beneficial for organ transplant recipients. The lead compounds were also subjected to numerous in vitro inhibition, absorption, and pharmacokinetic studies as part of drug development.

In 2003, Changelian and co-workers reported success with experimental allograft rejection with CP-690,550 [36]. This study was followed by a series of clinical studies for the use of this drug in allograft rejection, initially in 2009 by Stephan Busque from Stanford and a group of investigators from academia and from Pfizer labs [47]. This publication was followed by a few other similar clinical trials, that appeared to come to a dead end, likely due to safety and efficacy concerns. Nevertheless, a contemporaneous review stated that “The success of CP-690,550 in animal organ transplant models makes prevention of allograft rejection the most likely application of JAK inhibitors” [48].

### JAK Inhibitors in Inflammatory Diseases

Since the focus of this perspective is the use of JAK inhibitors for IBD, I shall now discuss the development of JAK inhibitors for this indication. Since JAK3 is always paired with JAK1 and is solely associated with the γ_c_ cytokine receptor whose activation is associated with inflammation in addition to lymphoid development and homeostasis (Fig. 2), it was hypothesized that partial JAK1/JAK3 inhibition might be anti-inflammatory without being markedly immunosuppressive, prompting investigators to seek indications among the autoimmune diseases.

The first disease investigated was rheumatoid arthritis (RA) and the related autoimmune arthridities, due in part to its response to cytokine inhibitors such as the tumor necrosis factor (TNF) monoclonal antibody infliximab and related studies [49,50] suggesting that the pathogenesis of RA is in part cytokine mediated. Parenthetically, infliximab was one of the first approved therapeutic monoclonal antibodies, itself a paradigm shift [51]. Several preclinical studies supported the contribution of the JAK-STAT pathway to RA, such one by Ulf Müller-Ladner and collaborators [52] from Germany and Switzerland who reported that the synovium from joints of patients with RA expressed IL-4 STAT (STAT6), a finding supported by several subsequent publications reporting the expression of JAK-STAT-SOCS pathway components in the synovia of patients with RA. These preclinical studies culminated by the 2009 publication by Joel Kremer from Albany Medical College and a group of international collaborators who reported a Phase-IIa trial of CP-690,550 in patients with RA, the success of which led to numerous follow-on studies eventuating in the FDA approval in 2021 of CP-690,550, later called tofacitinib, for the treatment of RA. [53]

After this extensive buildup, we finally get to the primary topic, the treatment of patients with IBD. An earlier paradigm shift occurred with the discovery that monoclonal antibodies directed against the cytokine TNFα achieved remarkable success in the treatment of patients with IBD [54,55], suggesting that IBD was a result in part of dysregulated immunomodulatory mechanisms. Surprisingly, few early preclinical studies directly addressed the contribution of the JAK-STAT pathways toward IBD pathogenesis, although numerous inferential studies have been published. Among the data cited linking the JAK-STAT pathways to IBD pathogenesis includes the identification of JAK2, TYK2, and STAT3 as risk genes for IBD and the extensive involvement of JAK-STAT components in T-cell development and function [56,57]. Among the most compelling data are the severe enterocolitis that occurred after somatic knockout of STAT3 in mice [58] that was prevented by treatment with anti-Il-12 antibodies. Further data implicating JAK-STAT pathway in colitis pathogenesis is the observation by Suzuki et al. in Japan [59] that STAT3 and the endogenous JAK-STAT inhibitor CIS3/SOCS3 are induced in experimental colitis models. Perhaps extrapolating from the efficacy and safety of tofacitinib in the treatment of patients with RA [5] and the considerable basic data linking the JAK-STAT pathway with the regulation of immune function [60], Sandborn et al. published the first clinical trial of tofacitinib in patients with moderate-to-severe UC in 2012 [61], showing promising results. The demonstrated efficacy and safety of tofacitinib set off an explosion of interest in the efficacy of JAK inhibitors in the treatment of patients with IBD that will be covered in the accompanying publication [7].

**Fig. 3:**
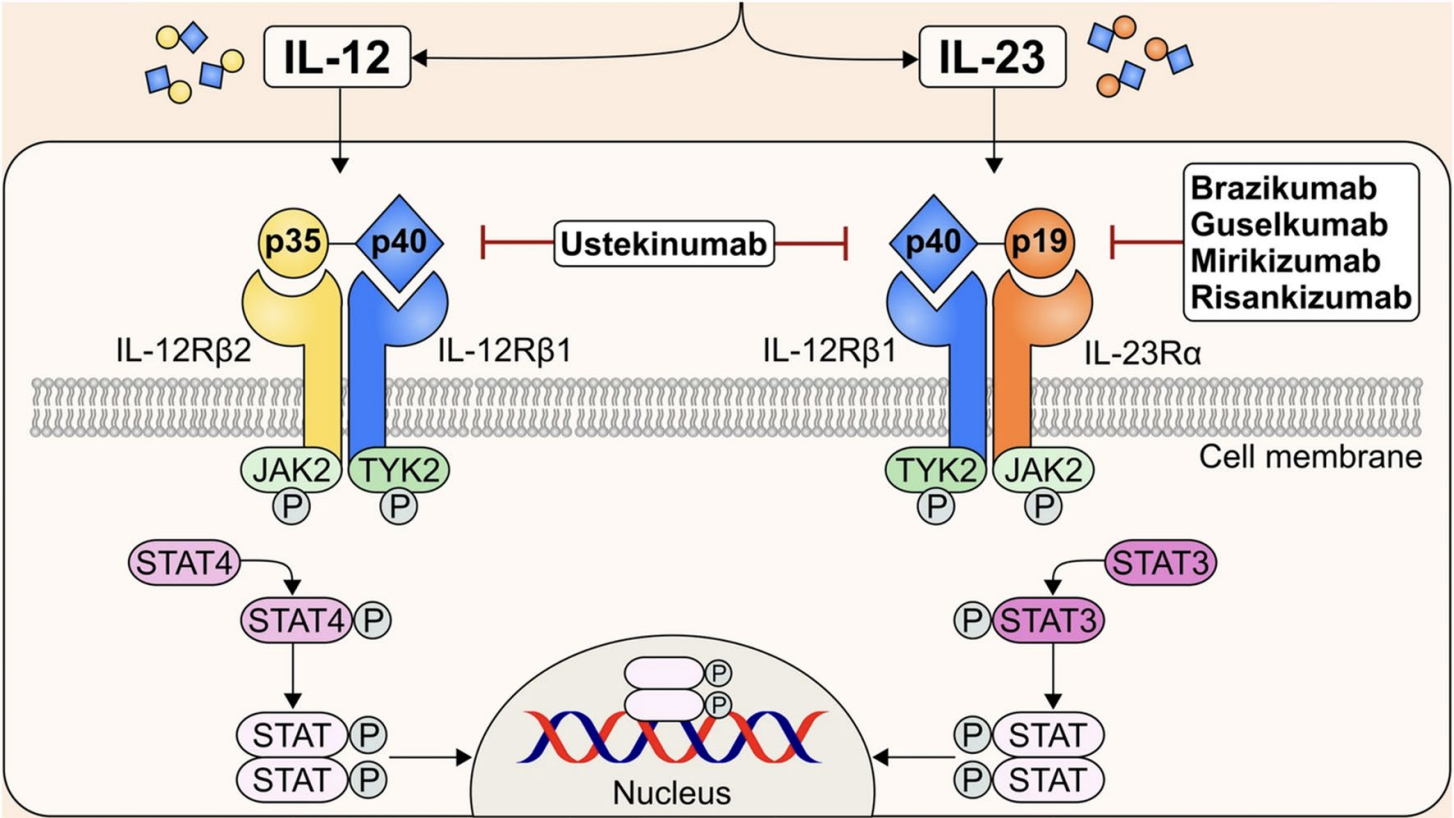
Signaling of IL-12/23 via the heterodimeric IL-12 and IL-23 receptors, TYK2, JAK2, and STAT3/4. Note that this system incorporates all the known IBD risk genes and is the target of the only approved biologic cytokine receptor inhibitors that signal through the JAK/STAT system (see text). Reprinted with permission from Ref. [71]

As previously discussed, tofacitinib, which is stated to be JAK1 and JAK3 selective, is believed to mostly inhibit the common γ-chain receptor family of cytokines, namely IL-2,IL-4,IL-7, IL-9, IL-13, IL-15, and IL-21 [57,62], that are not considered to be key to IBD pathogenesis [63,64]. Furthermore, since JAK1 is associated with multiple cytokine receptors and STATs (Fig. 2) and since tofacitinib also inhibits JAK2 to some extent, its activity against IBD is difficult to understand according to the current knowledge of JAK inhibitors. Interestingly, the heterodimeric IL-12/IL-23 receptors that signal through JAK2 and TYK2 have been successfully targeted in patients with IBD with biologics directed against IL-12/23 (ustekinumab) or IL-23 alone such as guselkumab, risankizumab, and many currently in development. This pathway, at least in theory, is not impacted by tofacitinib (Fig. 3). Finally, in contrast to RA, IL-6 has thus far proven an unsatisfactory target for IBD therapies [65]. Nevertheless, since JAK selectivity appears more closely linked to adverse effects than to clinical efficacy [63,66], and since JAK selectivity is subject to the multiple variables inherent in the selectivity assay used [33,44], the mechanism of action and clinical efficacy of the JAKinibs remains in large part notional, pending a more comprehensive understanding of their interactions with cellular signaling pathways.

## Final Words

The rapid progress of the development of the JAKinibs since their initial approval in 2008, with eleven currently FDA approved at the time of this writing, underscores that this drug class is in their infancy, with considerable potential yet to be revealed. An important caveat is that the “selectivity” of these inhibitors should not be regarded in the same context as conventional small molecule receptor inhibitors [63,67], since these drugs likely have complex actions over and above the kinase inhibition of one or more members of the JAK family, manifest in their approval for the treatment of diverse diseases other than RA and IBD: atopic dermatitis, myelofibrosis, polycythemia vera, graft-versus-host disease, alopecia areata, COVID-19, non-segmental vitiligo, psoriatic arthritis, ankylosing spondylitis, axial spondylarthritis, and juvenile idiopathic arthritis to date. A review of the simplified JAK-STAT signaling pathways reveals considerable redundancy and pleiotropism among the many cytokines, receptors, JAKs, STATS, and endogenous inhibitors that elude simple characterization of drug selectivity. A 2018 article [68] listed 41 cytokines, 20 cytokine receptors, four JAKs, seven STATs, and four SOCS family members, creating a bewildering number of targets and combinations thereof for these drugs, prompting Dr. Galien to remark in a recent publication [64]: “…it is difficult to build a case for one of the four JAKs, or even a combination of two of them, as a good or best target for IBD treatment.” Interestingly, many of these elements appear to be under the control of super-enhancers, regulators of expression of an array of genes over space and time that in lymphocytes were altered by the JAKinib tofacitinib [69], further hinting at the complexity of the mechanism of action these simple-appearing small molecules. Indeed, the original studies of Uckun et al. [35] in which selectivity was measured in vitro with a single simple kinase assay likely provided a skewed assessment of selectivity that resulted in the promotion of drugs whose clinical development was eventually halted presumably due to disappointing results. A more sophisticated and possibly nuanced approach was published by Traves et al. [63] who tested the effect of five JAKinibs on three classes of immunocytes stimulated by a variety of cytokines to phosphorylate different STATs, concluding that: “…the inhibitory effect of each JAKinib is dependent on the specific cytokine stimulus, STAT substrate, and cell type, so the association of pathway inhibition with clinical impact would require cell-specific cytokine evaluation.” Given the severity of the diseases associated with malfunctioning of the JAK-STAT pathway [70], including myeloproliferative disorders, numerous solid and hematologic tumors, severe combined immunodeficiency, asthma, and myocardial hypertrophy, I must admire the courage and vision of the investigators who originally trialed these drugs in humans, since their foresight set off a therapeutic revolution that is just starting to gain traction.

## Data Availability

No datasets were generated or analyzed during the current study.
